# Respiratory Mechanics in Acute Respiratory Distress Syndrome: A Quality Improvement Based Registry Project

**DOI:** 10.1186/2197-425X-3-S1-A507

**Published:** 2015-10-01

**Authors:** L Chen, GQ Chen, C Martins, K Porretta, O Shklar, P Greco, H Every, M Xu, JX Zhou, L Brochard

**Affiliations:** University of Toronto, Interdepartmental Division of Critical Care Medicine, Toronto, Canada; St. Michael's Hospital, Keenan Research Centre and Li Ka Shing Insitute, Department of Critical Care, Toronto, Canada; Beijing Tiantan Hospital, Department of Critical Care Medicine, Beijing, China; St. Michael's Hospital, Department of Respiratory Therapist, Toronto, Canada

## Introduction

The amount of pathophysiological impairment in patients with acute respiratory distress syndrome (ARDS) is variable and applying the same ventilator regimen to every patient is questionable. Monitoring of respiratory mechanics for the lung and chest wall allows an individualization of ventilator settings with a potential benefit for the patient. a registry with a large sample size will elicit helpful epidemiological information and may inform future recommendations. We therefore proposed a quality improvement (QI) program constituted by systematic assessment of respiratory mechanics and gas exchange response. the collected data are then introduced into a registry. We report here the preliminary results.

## Objectives

The QI program aims at facilitating the integration of respiratory mechanics monitoring in ventilatory management. the primary objective of the registry is to investigate the epidemiology of abnormalities in respiratory mechanics in ARDS patients.

## Methods

Two ICUs in Toronto and one ICU in Beijing have initiated this multi-center project. Patients admitted to the ICUs who meet the Berlin definition of ARDS are eligible[1]. Placement of an esophageal catheter is considered when PaO2/FiO2 ≤200. Systematic measurement are performed by the clinicians, including respiratory mechanics, lung and chest wall mechanics, oxygenation response to PEEP, and alveolar derecruitment using a simplified decremental PEEP maneuver[2]. After obtaining the first measurement, a comparison of ventilator settings before and after measurements is conducted and we also report the epidemiology of respiratory mechanics abnormalities observed.

## Results

50 ARDS patients have been enrolled (Men: 34, Age: 52 ± 22 years, SOFA: 12 ± 5): 7 patients (14%) had mild ARDS, 33 (66%) moderate ARDS, and 10 (20%) severe ARDS. Esophageal pressure was measured in 46 patients with an occlusion test ratio (ΔPaw/ΔPes) at 0.94 ± 0.18. in 39 patients (78%), the ventilator settings were changed according to measurements, often by limiting VT and PEEP. the physiological variables in respiratory mechanics are described in Table 1. We found that on average 74% of the driving pressure to distend the respiratory system was due to the lung, but with extremes from 48% to 92%. the ICU mortality was 34%.

**Table 1:** *Epidemiology of respiratory mechanics (N = 50).*

Variable	Values	Extreme	Unit
Pplat	25 (21-30)	13-37	cmH_2_O
PEEPtot	12 (10-15)	5-19	cmH_2_O
Vt/PBW	6.3 (6.0-6.7)	3.0-7.8	ml/kg
Pdriv	13 (9-16)	7-25	cmH_2_O
Ers	30 (22-37)	16-74	cmH_2_O/L
EL/Ers	74 (65-80)	48-92	%
PaO_2_/FiO_2_, low PEEP* PaO_2_/FiO_2_, high PEEP*	143 (103-190) 142 (118-200)	60-311 58-282	mmHg mmHg
Vder	120 (69-200)	0-426	ml

Values were described as medians [interquartile ranges], and the extreme was reported as minimum-maximum.

Pplat: plateau pressure; PEEPtot: total PEEP; Vt/PBW, tidal volume per predicted body weight; Pdriv: driving pressure of respiratory system; Ers: elastance of respiratory system; EL/Ers: the ratio of lung elastance to respiratory system elastance; Vder: derecruited volume.

* Assessment of oxygenation response by changing PEEP in 3-5 cmH_2_O.

## Conclusions

A QI program integrating respiratory mechanics monitoring with ventilator management is feasible, lead to individual adaptations and can provide epidemiological information for better understanding respiratory mechanics in ARDS patients.
